# Predicting demographics from meibography using deep learning

**DOI:** 10.1038/s41598-022-18933-y

**Published:** 2022-09-20

**Authors:** Jiayun Wang, Andrew D. Graham, Stella X. Yu, Meng C. Lin

**Affiliations:** 1 Vision Science Graduate Group, Herbert Wertheim School of Optometry and Vision Science, University of California, 360 Minor Hall, MC#2020, Berkeley, CA 94720-2020 USA; 2 Department of Electrical Engineering and Computer Sciences, University of California, Berkeley, USA; 3 Clinical Research Center, Herbert Wertheim School of Optometry and Vision Science, University of California, Berkeley, USA; 4 International Computer Science Institute, Berkeley, USA

**Keywords:** Biomarkers, Predictive markers, Diseases, Eye diseases, Health care, Health policy, Medical imaging

## Abstract

This study introduces a deep learning approach to predicting demographic features from meibography images. A total of 689 meibography images with corresponding subject demographic data were used to develop a deep learning model for predicting gland morphology and demographics from images. The model achieved on average 77%, 76%, and 86% accuracies for predicting Meibomian gland morphological features, subject age, and ethnicity, respectively. The model was further analyzed to identify the most highly weighted gland morphological features used by the algorithm to predict demographic characteristics. The two most important gland morphological features for predicting age were the percent area of gland atrophy and the percentage of ghost glands. The two most important morphological features for predicting ethnicity were gland density and the percentage of ghost glands. The approach offers an alternative to traditional associative modeling to identify relationships between Meibomian gland morphological features and subject demographic characteristics. This deep learning methodology can currently predict demographic features from de-identified meibography images with better than 75% accuracy, a number which is highly likely to improve in future models using larger training datasets, which has significant implications for patient privacy in biomedical imaging.

## Introduction

The Meibomian glands of the human eyelid secrete lipid-rich meibum that during blinking forms a thin film on the surface of the tears^1,2^ that serves to inhibit evaporation of the tear aqueous and stabilize the tear film by reducing surface tension^3,4^. Dysfunction of the Meibomian glands leading to insufficient or poor quality lipids is a primary cause of dry eye (DE)^5,6^, a globally impactful and highly prevalent ocular surface disease^7^. Infrared meibography is the biomedical imaging of the Meibomian glands, viewed by everting the eyelids, using a thermographic camera. Meibography has been increasingly used in recent years for clinical diagnosis and management of Meibomian gland dysfunction (MGD) as well as in clinical research on MGD and DE; however, there are few studies that have examined how the detailed morphological structure of these glands relates to the signs and symptoms of MGD and DE^8–10^. Furthermore, it is unknown how the detailed morphology of the Meibomian glands relates to subject demographic characteristics such as age, gender, and ethnicity—all of which are well-documented factors in the prevalence and severity of MGD and DE^11–16^.

There are a number of studies to date that have employed traditional statistical techniques to examine broad, global assessments of the Meibomian glands such as the overall percent area of gland atrophy (e.g., the 4-level meiboscore of Arita^17^, the 5-level score and software-based atrophy area of Pult^18^) and their relationships to various subject characteristics. With few exceptions, however, the detailed, local morphological characteristics of the glands in meibography images (e.g., length, width, tortuosity, local contrast) have not been well studied with respect to subject characteristics or clinical outcomes. A major impediment to detailed morphological analysis of meibography images and to its use in both research and clinical eyecare has been the technical difficulty and time-consuming nature of quantifying local meibography features^19^. In a previous work we developed a deep learning model that proved capable of quickly and automatically identifying and quantifying eight different metrics describing both global and local morphological features in novel meibography images with good accuracy^20^. In this study we will build on this work by training a supervised machine learning model to identify and quantify the morphological features observed in de-identified meibography images and then use these images and image-derived metrics to predict the demographic characteristics of the subjects who provided them.

The significance of attempting to predict subject demographics from meibography images is twofold. First, it offers an alternative approach to traditional associative statistical modeling for determining whether the morphology of the Meibomian glands differs depending on age, gender, or ethnicity—all known factors in MGD and DE. Rather than testing a null hypothesis under certain assumptions, we train the machine learning model not only to learn to use meibography image features to predict subject demographic characteristics but to reveal what the most highly weighted image features were in contributing to this prediction. This could shed further light on the etiology of MGD at a more detailed level, and possibly reveal novel relationships.

Second, de-identified biomedical imaging is not currently considered Protected Health Information (PHI), and is therefore not subject to the strict regulations on its use, sharing, storage and transmission^21–23^. These regulations, however, are in active debate and are likely to evolve rapidly, as ocular features such as retinal vein patterns, eye movements, and iris patterns have been proven to provide unique biometric “fingerprints” that can be used to identify individuals with a high level of accuracy^24–26^. It seems reasonable, given the highly detailed morphology revealed in meibography images, that meibography could be developed into a biometric identifier as well, thus requiring far greater patient safety and privacy protections—even for de-identified images—than it is subject to today. The field of artificial intelligence and machine learning is evolving rapidly and its capabilities ever-expanding. If we can train a machine learning model now to take de-identified meibography images as input, and based solely on the detailed morphology revealed in those images, reconstruct some characteristics of the subjects that provided them, it is very easy to imagine with larger training datasets and improved models, being able in the near future to reveal a patient’s individual identity.

## Methods

### Development and evaluation datasets

This study utilized a meibography image dataset from a previously published work^20^ along with corresponding subject demographic information for deep learning algorithm development and evaluation.

#### Subject recruitment and imaging

Adult human subjects (age $\ge$ 18 years) were recruited from the University of California (UC), Berkeley campus and surrounding community for single-visit ocular surface evaluations at the UC Berkeley Clinical Research Center during the period from 2012 to 2017. Eligible subjects were free of any eye conditions contraindicating meibography, not currently taking medications with effects on the anterior eye or adnexa, and with no history of ocular surgery. The research protocol adhered to the tenets of the Declaration of Helsinki and was approved by institutional review board (UC Berkeley Committee for Protection of Human Subjects). Informed consent was obtained from all subjects after being informed of the goals, procedures, risks, and potential benefits of the study. Meibography images of the upper eyelids for both eyes were captured with the OCULUS Keratograph 5M (OCULUS, Arlington, WA), a clinical instrument that uses an infrared light with a wavelength of 880 nm for Meibomian gland imaging^27^. During image capture, the ambient light was off with the subject’s head positioned on a chin rest and forehead strap apparatus. A total of 750 images were collected and pre-screened to rule out images that did not capture the entire upper eyelid (61 images or 8.90%); the remaining 689 images were used in the analysis.

#### Demographics

**Figure 1:**
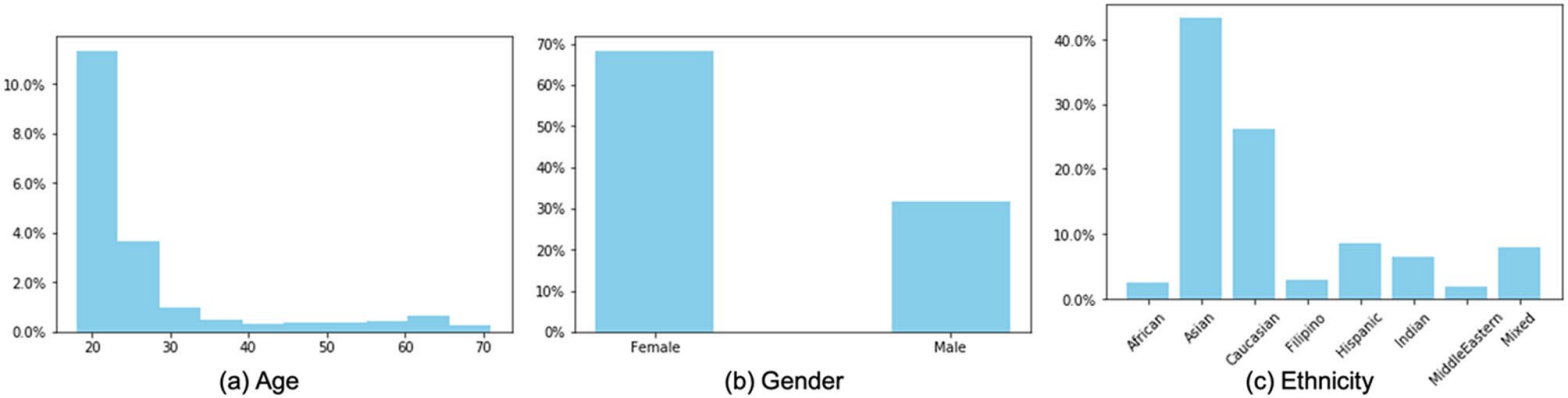
Histograms (in percentage) of the demographic features of our meibography image dataset.

Subject demographics were documented during the visit. Three demographic characteristics were studied in this work, namely age, gender and ethnicity. Histograms depicting the distributions of these demographic features are presented in Fig. 1. The lack of sufficient subjects of some ethnicities for adequate training of the model allowed us only to make accurate predictions for our two largest groups: Caucasians and Asians. The total number of images used for ethnicity prediction is thus 421, while all 689 images were used for age and gender prediction.

#### Morphological features

**Figure 2:**
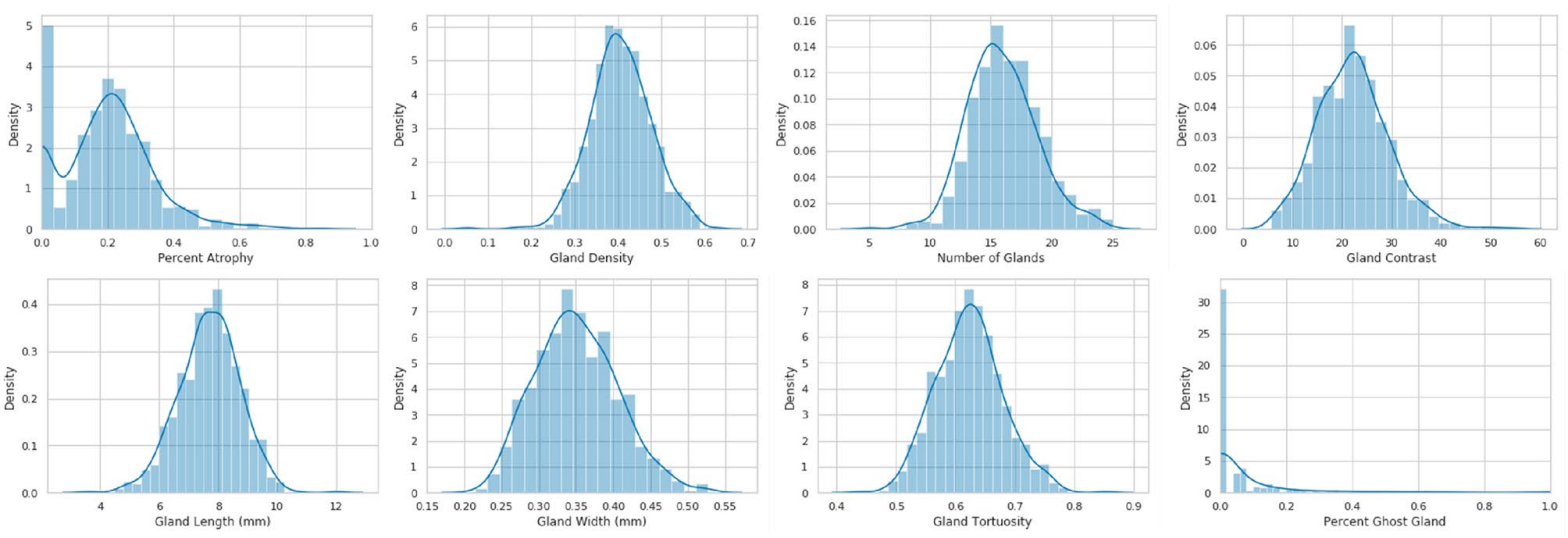
Histograms and density plots of morphological features from meibography images.

The development of an interpretable deep learning model for predicting demographic characteristics requires morphological features such as gland length and tortuosity as data sources. Eight morphological features were quantified for each meibography image as in our previous work: number of glands, gland density, percent area of gland atrophy, gland local contrast, gland length (mm), gland width (mm), gland tortuosity and percentage of ghost glands^20^. Histograms of these morphological features are presented in Fig. 2.

#### Data partitioning

Meibography images were partitioned into two mutually exclusive subsets for training and evaluating the deep learning model. Images collected from years ranging from 2015 to 2017 were combined to constitute the development set, while those collected from years ranging from 2012 to 2013 were combined to constitute the evaluation set. All images were taken with the same instrument under the same protocol. The development set was further divided randomly into 2 subsets for training and validating the model. Specifically, the validation set was used to fine-tune the model hyperparameters (e.g., model learning rate) for the model that was trained on the training set. The evaluation set was used for evaluating and testing the performance of the model. Subject demographics stratified on development and evaluation datasets are shown in Table 1. Different subsets had similar demographic feature distributions, so that the distributional shift between the training and evaluation sets was minimized.

**Table 1:** Subject demographics of the meibography image dataset used in the study.

	Development		Evaluation
	Train	Tune	
Images (n)	389	97	203
**Subject demographics**			
Subjects (n)	260	94	109
Mean (SD) age (years)	27.8 (13.1)	27.0 (11.5)	27.9 (12.7)
% female subjects	69.6	66.0	69.4

### Algorithm design and training

**Figure 3:**
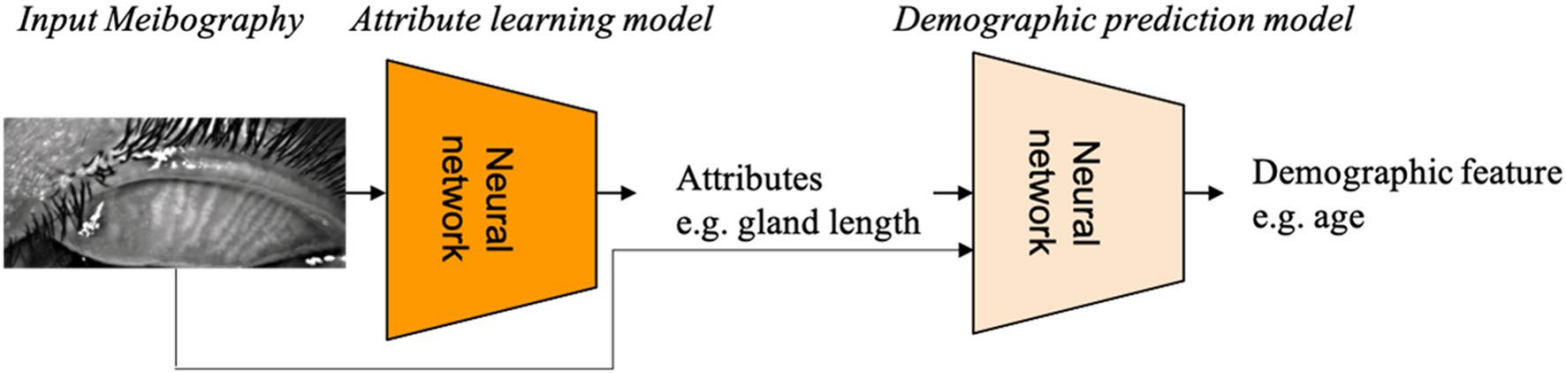
Overall pipeline of the interpretable deep learning model for predicting demographics from images.

The overall goal is to design an interpretable deep learning model that can predict the demographic characteristics of a subject. Interpretability requires the model to be able to identify the most highly weighted morphological features used by the algorithm to predict the demographic characteristics of a subject directly from their meibography image. A two-stage model was designed with a first stage attribute learning model to identify and quantify morphological features from input meibography images, and a second stage demographic prediction model to predict subject demographic features from meibography images and corresponding first-stage morphological features. Figure 3 depicts the overall pipeline.

#### Deep attribute learning

In the first stage, a deep learning model was developed to predict and quantify the morphological features of a given meibography image (first part of Fig. 3). The primary goal of the attribute learning model is to provide value ranges rather than exact values of morphological features for the final demographic predictions. There are two underlying reasons for this: (1) predicting coarser value ranges is easier than predicting precise values for the deep learning model, especially since the dataset (689 images in total) was not sufficiently large-scale to learn precise morphological feature values. (2) Morphological attribute prediction was an intermediate result, with the major purpose of interpreting relationships between demographic features and morphological features. Predicting value ranges is adequate for the purpose. For example, it would be acceptable for predicting gender to find that females exhibit a high probability of having > 15 glands rather than a high probability of having exactly 16 glands. Therefore, our first stage deep learning model predicts morphological features to fall within ordinal ranges (or, in the case of ghost gland percentage, binary classes).

**Figure 4:**
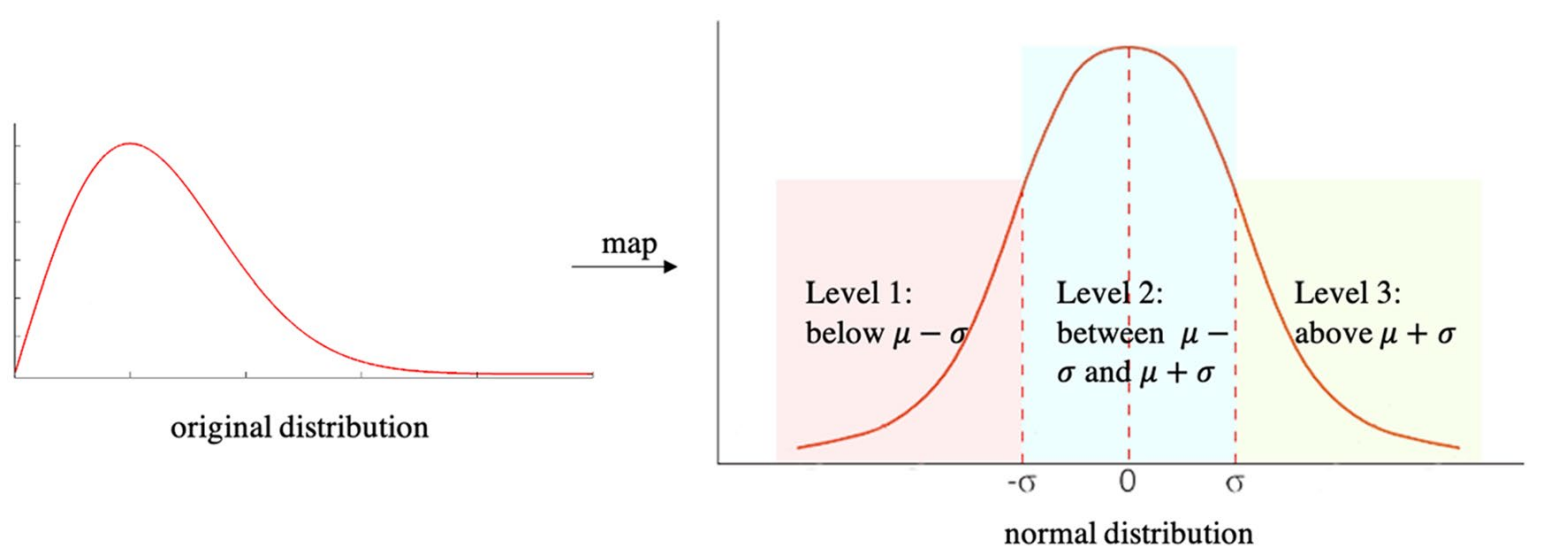
Distribution mapping for the deep attribute learning model. The original distribution of a morphological feature is mapped to a normal distribution. The deep attribute learning model predicts if a morphological feature value is below $\mu -\sigma$, between $\mu -\sigma$ and  + σ, or above $\mu +\sigma$, where $\mu$ and $\sigma$ refer to the mean and standard deviation of the original morphological feature value distribution.

The morphological attribute learning model specifically predicts a ternary level rather than an exact numerical value for each morphological feature. As depicted in Fig. 4, the model predicts each morphological feature value to fall below $\mu -\sigma$ (level 1), between $\mu -\sigma$ and $\mu +\sigma$ (level 2), or above $\mu +\sigma$ (level 3), where $\mu$ and $\sigma$ refer to the mean and standard deviation of the morphological feature predicted value distribution. Table 2 provides the $\mu$ and $\sigma$ for all morphological features investigated. In the case of percentage of ghost glands, 77.1% images (or 531 images) have 0 ghost glands. Therefore, for the percentage prediction, a binary class was used (percentage of ghost glands = 0 or > 0).

**Table 2:** Means and standard deviations of morphological features.

	Percent atrophy (%)	Gland density (%)	# of glands	Gland local contrast*	Gland length (mm)	Gland width (mm)	Gland tortuosity (%)	% ghost glands (%)**
Mean	19.7	40.7	15.9	22.2	7.7	0.35	63.6	7.8
Standard deviation	13.8	7.1	2.8	7.2	1.0	0.05	10.6	16.9

*Measured in pixel intensity of the meibography image. The lowest intensity the sensor could detect was 0 and the highest was 255.

**For the percentage of ghost glands prediction, a binary classification was used (= 0, > 0).

Specifically, for each morphological attribute (e.g., gland length), a meibography image was fed to a ResNet18 (a residual neural network of 18 convolution layers)^28^ to obtain a 64-dimensional vector. The vector was made available by directly adding a fully connected layer after the last convolution layer of ResNet18. The 64-dimensional feature vector was then fed to another fully connected layer for classifying the corresponding attribute (e.g., ternary level of the gland length). The process was the same for all 8 morphological attribute prediction models, meaning that for each meibography image, there were 8 64-dimensional vectors with each one indicating the corresponding morphological attribute.

#### Demographic feature prediction

In the second stage, a deep learning model was developed to predict demographic features from both meibography images and corresponding attributes from the attribute learning model in stage one (second part of Fig. 3). Specifically, a given image was input to ResNet18^28^ to obtain a 64-dimensional vector. The vector can be considered as an embedding that encodes information of the image. The vector was combined with 8 predicted vectors of morphological features from the stage one deep attribute learning model. All vectors are of the same dimension. The combined 9 vectors were input to a fully convolutional layer for predicting the demographic features.

Among the three demographic features to be predicted, gender and ethnicity are categorical, while age is continuous numerical. Following Dana et al.^29^ based on dry eye prevalence, subject age was stratified into three categories: (1) ≤ 39 years old, (2) > 39, < 50 years old, and (3) ≥ 50 years old.

The final output of the demographic prediction model can be interpreted by analyzing the learned coefficients of the morphologic features used to predict the demographic characteristics. Higher coefficient values indicate a stronger weighting of a morphological feature in predicting a demographic feature.

### Evaluation metrics

The model was trained on the training set with varying hyperparameters (e.g., different learning rates) and the highest performance model on the validation set was selected for final evaluation on the evaluation set. The highest performance models were selected for attribute learning and demographic prediction, and were evaluated by their classification accuracy.

#### Classification evaluation with tolerance threshold

**Figure 5:**
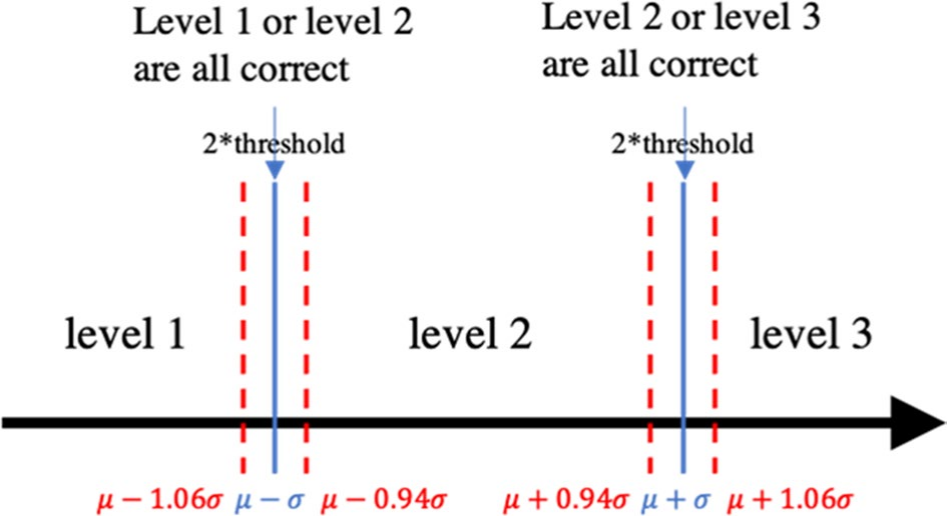
The relaxed morphological feature classification evaluation rule with the tolerance threshold. The tolerance threshold was set at 0.06$\sigma$, and classifying an image with percent atrophy $\mu -1.06\sigma$ to $\mu -0.94\sigma$, $\mu -0.06\sigma$ to $\mu +0.06\sigma$, and $\mu +0.94\sigma$ to $\mu +1.06\sigma$ either to its ground-truth or adjacent level were considered as a correct predictions.

The evaluation technique was used for evaluating deep attribute learning performance. As described in the previous section, the stage one deep attribute learning model predicts the trinary level of each morphological feature (or binary, for percentage of ghost glands). However, near the transition limits of different levels ($\mu -\sigma$ and $\mu +\sigma$), the morphological features may be very similar and difficult to classify. A similar technique described in Wang et al.^30^ was applied here. A tolerance threshold near the grading transition limit was necessary. As illustrated in Fig. 5, the tolerance threshold was set at 0.03$\sigma$, and classifying morphological feature values within $\mu -1.06\sigma$ to $\mu -0.94\sigma$, and $\mu +0.94\sigma$ to $\mu +1.06\sigma$ either to their ground-truth or adjacent level were both considered as correct predictions. Note that the tolerance threshold does not apply to predictions of the percentage of ghost glands as that is a binary classification.

#### Five-fold cross-validation

For evaluating both attribute learning and demographic prediction performance, in addition to reporting classification accuracy on the evaluation set with the best performing model on the validation set, five-fold cross-validation accuracy is also reported. First, the entire dataset (including both development and evaluation subsets) was randomly partitioned into 5 folds. Second, 5 iterations of training and evaluation were conducted. At each iteration, 4 folds were used for training and the remaining fold for evaluation. The mean and standard deviation of the classification accuracy on each fold were reported as five-fold cross-validation accuracy.

## Results

### Attribute prediction performance

Table 3 reports the performance of the stage one attribute prediction accuracy for morphological features. Specifically, model accuracies on the evaluation set and five-fold cross-validation are reported. For attribute prediction (including percent atrophy, gland density, number of glands, gland local contrast, gland length, gland width, gland tortuosity and percentage of ghost glands), the model achieved 76.5% accuracy on the evaluation set, and 76.6% average accuracy for five-fold cross-validation. The average standard deviation of the five-fold cross-validation was 4.0%.

**Table 3:**
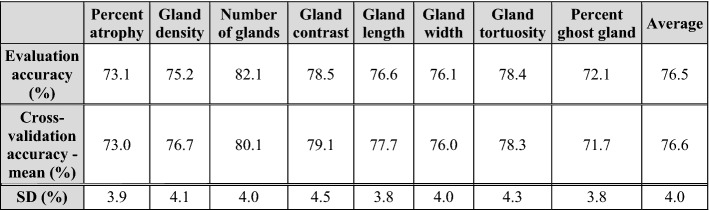
Classification accuracy for morphological feature prediction of the attribute learning model. The model predicts the gland morphological features with an average accuracy of 77%.

Note that the second row reports accuracies on the evaluation set and the last two rows reports means and standard deviations of accuracies on the five-fold cross-validation set.

### Demographic prediction performance

For each subject demographic feature, classification accuracy of the stage two demographic prediction model is reported. Similarly, accuracies on the evaluation set and five-fold cross-validation are reported. Additionally, the coefficients for Meibomian gland morphological features were also analyzed by ranking coefficients and identifying the most highly weighted morphological features in the demographic predictions. The average values of morphological features in the different demographic groups were then compared. For each split in the five-fold cross-validation, morphological features ranked from most highly weighted to least were recorded. For both age and ethnicity, the 2 most important features were observed to be consistent for every split. Average values for the top 2 most important morphological features stratified on demographic group are reported below.

#### Age

The five-fold mean (SD) classification accuracy for age was 75.7 (4.5)% (Table 4 upper). The sensitivities and specificities of the models for distinguishing each age group from the other two were as follows: (a) for [age ≤ 39 years] vs. [age > 39  years], sensitivity and specificity were 86.1% and 89.2%, respectively; (b) for [39  years  < age < 50  years] vs. [age ≤ 39  years & age ≥ 50  years], sensitivity and specificity were 70.1% and 89.4%, respectively; (c) for [age ≥ 50  years] vs. [age < 50  years], sensitivity and specificity were 71.1% and 94.7%, respectively. Coefficient analysis showed the two most important morphological features for determining age were percent area of gland atrophy and percentage of ghost glands (Table 4 lower). For the three age groups from youngest to oldest, the average percent area of atrophy increases from 18.1 to 25.2 to 33.6%, while the average percentage of ghost glands increases from 5.6 to 14.2 to 28.7%. Older subjects exhibited a higher percent area of atrophy and a higher percentage of ghost glands compared with younger subjects.

**Table 4:**
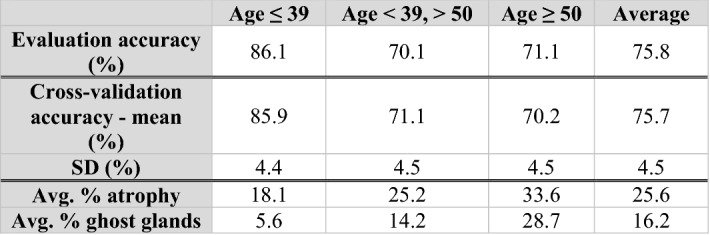
Age prediction results. The model predicts the subject age with an average accuracy of 77%, and the two most important morphological features are percent area of gland atrophy and percentage of ghost glands. First 4 rows: classification accuracy for subject age prediction of the demographic feature prediction model.

The second row reports accuracies on the evaluation set and the last two rows report means and standard deviations of accuracies on the five-fold cross-validation set. Last two rows: Average values for the top 2 most highly weighted (largest model coefficients) morphological features stratified on age group.

#### Gender

The five-fold mean (SD) classification accuracy for gender was 56.5 (5.0)% which is close to a random guess accuracy of $50\%.$ Therefore, gender could not be accurately predicted from meibography images with the proposed deep learning model, nor could important morphological differences be identified.

#### Ethnicity

As shown in the histogram of ethnicity (Fig. 1c), although the entire dataset was comprised of subjects of eight different ethnicities, most ethnicities did not have sufficient sample sizes for trustworthy predictions to be obtained. Therefore, predictions were only conducted for Asian and Caucasian subjects (75% of all subjects). The five-fold mean (SD) classification accuracy for ethnicity (predicting if the subject is Asian or Caucasian) was 85.7 (4.5)% (Table 5 upper). The sensitivities and specificities of the models for distinguishing Asians and Caucasians were 86.4% and 85.1%, respectively. Coefficient analysis showed the two most important Meibomian gland morphological features for determining ethnicity were gland density and percentage of ghost glands (Table 5 lower). Asian subjects exhibited approximately 2.8% greater gland density than Caucasians. Asian subjects exhibited 2.6% fewer ghost glands than Caucasians.

**Table 5:**
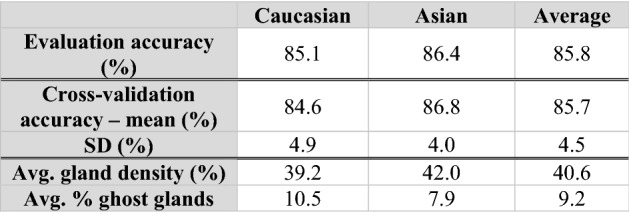
Ethnicity prediction results. The model predicts the subject ethnicity with an average accuracy of 86%, and the two most important morphological features are gland density and percentage of ghost glands. First 4 rows: classification accuracy for subject ethnicity prediction of the demographic feature prediction model.

The second row reports accuracies on the evaluation set and the last two rows report means and standard deviations of accuracies on the five-fold cross-validation set. Last two rows: average values for the top 2 most highly weighted (largest model coefficients) morphological features stratified on ethnic group.

**Figure 6:**
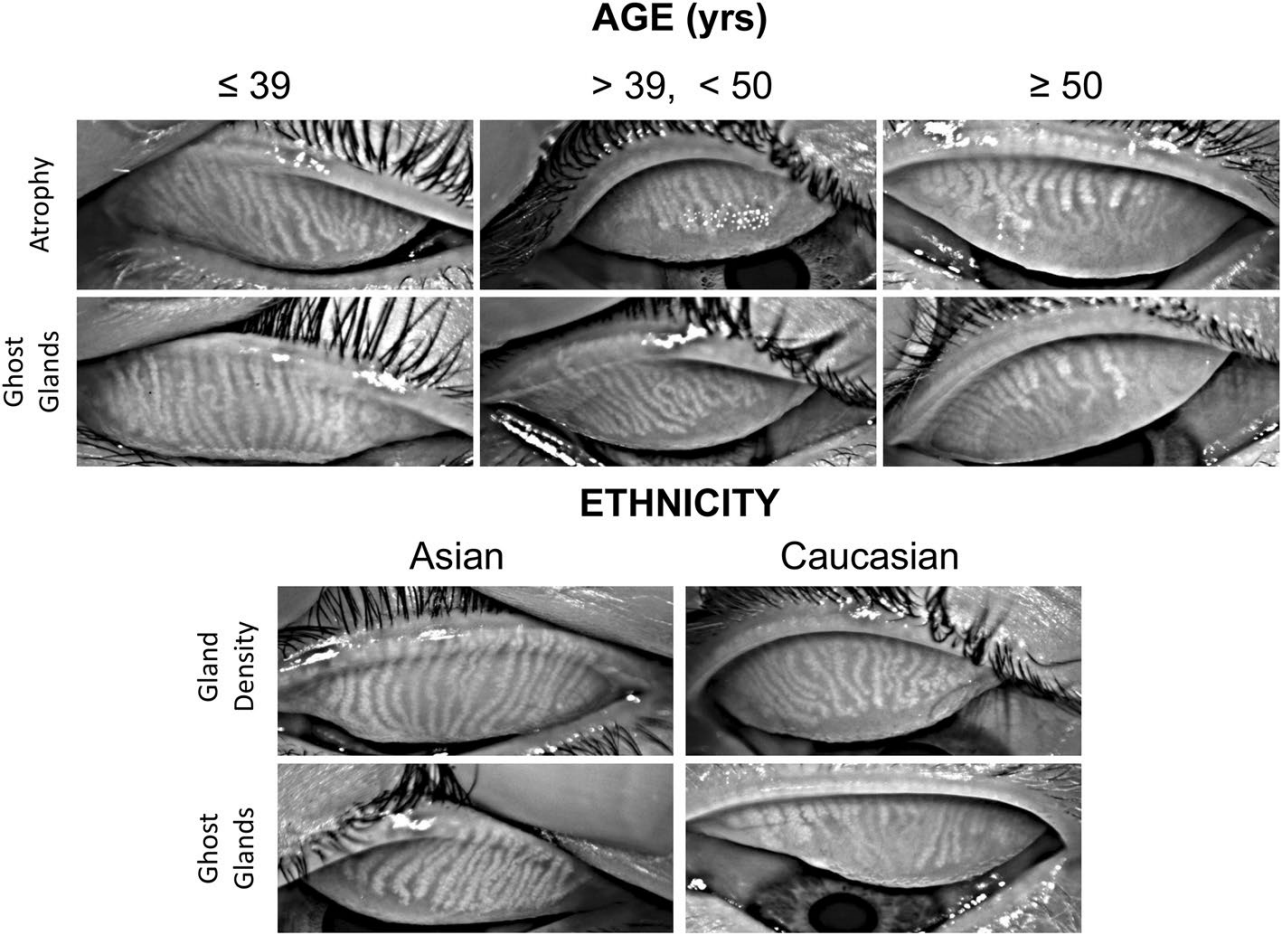
Meibography images stratified on age and ethnic group, illustrating the most highly weighted Meibomian gland morphological features used by the prediction model. Older subjects exhibited more MG atrophy and ghost glands. Caucasian subjects exhibited lower gland density and more ghost glands compared with Asian subjects.

Representative meibography images stratified on age and ethnic group, illustrating the most highly weighted Meibomian gland morphological features used by the prediction model, are shown in Fig. 6.

#### Ablation study on the attribute module and image as additional input

The demographic prediction model takes both learned MG attributes and the meibography image itself as inputs. A post-hoc ablation study was conducted to understand the effects of each input on the final classifications. As reported in Table 6, removing either the attribute module or the full image as additional input leads to lower age and ethnicity classification accuracy.

**Table 6:** Ablation studies of the attribute learning module and using images as additional inputs in the demographics prediction model. The proposed model with both attribute learning model and images as additional inputs leads to the highest prediction accuracy.

Classification accuracy (%)	Attribute only	Image only	Attribute + image
Age	68.7	72.4	75.8
Ethnicity	79.2	83.0	85.8

For age and ethnicity classification, removing either the attribute module or the image as additional input leads to decreased classification accuracy.

#### Grad-CAM visualization

**Figure 7:**
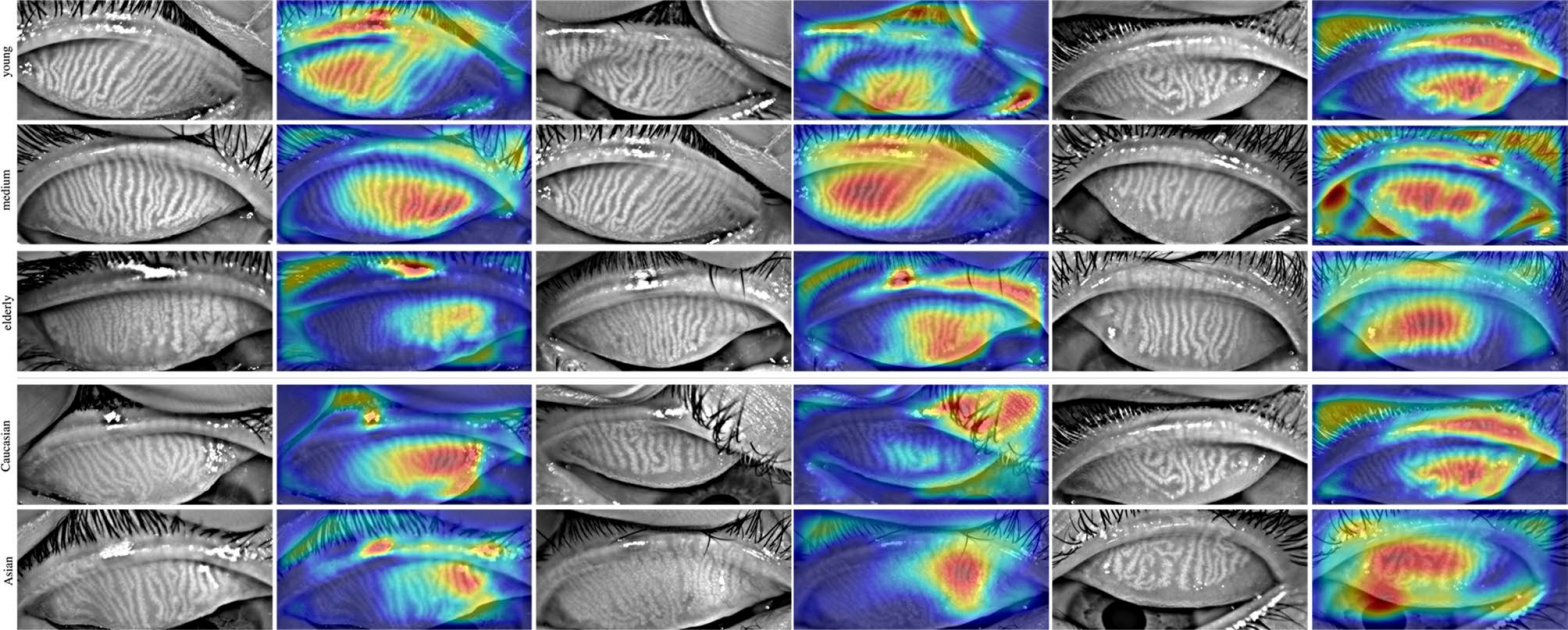
Grad-CAM was applied to the demographic prediction model to visually understand the important regions of the meibography images that contributed to the final classification. The most important visual regions strongly trended towards the glandular image areas.

Gradient-weighted Class Activation Mapping (Grad-CAM)^31^ is a technique that produces a visual explanation for decisions from convolutional neural networks. We applied Grad-CAM to understand the important regions of the meibography images that contributed to the final classifications. As shown in Fig. 7, the most important visual regions strongly trended toward the glandular image areas. The Grad-CAM results provide an additional, qualitative measure of confidence that the prediction model was more strongly weighting image features associated with the MG regions, as opposed to image artifacts with no clinical importance.

#### Hardware and inference speed

The networks were trained and evaluated on a single NVIDIA GeForce GTX 2080 Ti GPU with NVIDIA CUDA v11.0 (NVIDIA, Santa Clara, CA, USA). The average processing time per meibography image was approximately 0.21 s, allowing for the processing of 1000 images in approximately 3.5 min.

## Discussion

The work presents an interpretable deep learning model to predict demographics from meibography images. The proposed approach makes the following two contributions: (1) using deep learning models to find morphological features predictive of demographics offers an alternative to traditional associative modeling and may reveal new relationships; (2) the proposed approach investigates an early stage of the technology that could be used to develop meibography images into a biometric fingerprint capable of identifying individuals.

Previous studies have explored the associations between imaging of the eyelid and systemic conditions such as diabetes mellitus^32^ that are thought to increase the risk of DE^33^. The symptoms of MGD and DE are well known to be associated with other subject-level characteristics^14–16^, including age^34,35^ and ethnicity^36^. The subjectively graded % area of gland atrophy in meibography images is thought to increase with age^11^, and ethnic differences in subjectively graded meibography have been documented in a pediatric cohort^13^. To our knowledge, very little other information directly relating meibography image features and demographics is currently available. Given the likely links between eyelid condition as revealed in biomedical imaging, subject-level characteristics, and symptoms of MGD and DE, in this study we explored associations between subject demographics and specific Meibomian gland morphological features, as well as image-level meibography features.

The results showed that older subjects had a higher percent area of gland atrophy and a higher percentage of ghost glands. Previous works^37,38^ have identified age-related changes Meibomian gland structure and function, including changes to the acini, loss of progenitor stem cells, abnormal meibum secretion, and MGD. The deep learning method also identified age-related changes with a new focus on changes in gland morphology which was quantitatively analyzed directly from meibography images.

The deep learning model was unable to predict gender with high accuracy. Previous works^12^ however identified differences in the anatomic lid margin and gland morphology in different gender groups. It is possible that the inability of the deep learning model to accurately predict gender from meibography images was due to an insufficient number of subjects to train the model to identify subtle intergroup differences. It may also be the case that the proposed deep learning method itself was not amenable to identifying gender groups, and may require alternative machine learning architectures. Finally, it may be that meibography images alone do not provide enough information for accurate gender prediction, and may need to be combined with clinical signs such as tear film breakup time in order for the model to recognize gender differences. Future work is warranted to determine whether meibography images encode sufficient gland morphological information for revealing subject gender.

As for ethnicity, Caucasians subjects exhibited a higher percentage of ghost glands and lower gland density compared with Asian subjects. Kim et al.^13^ observed ethnic differences between Asians and Caucasians in Meibomian gland morphological patterns in the pediatric population by qualitatively identifying MG morphological changes. They found that the presence of gland shortening is more frequent in Asians while gland tortuosity is more frequent in Caucasians. The major differences compared to this study lie in efficiency and extensibility. In the Kim et al. study, 70 meibography images were manually annotated for morphological features, compared with this study which employed a deep-learning-based model to automatically analyze morphological features directly from meibography images. The present study was also able to utilize a much larger meibography image dataset ($n=689$), and no additional manual annotation is needed for novel images.

In the United States, health care providers and researchers are held accountable for the privacy and security of protected health information (PHI) and individually identifiable health information^39^. However, the frequency and magnitude of health care data breaches continues to climb^40^, with significant impacts for patients in the areas of identity theft, faulty treatment, insurance coverage, job security, financial well-being, and mental health^41,42^. While the majority of a person’s medical and health information is considered PHI and thus subject to strict regulations on its use, storage, and dissemination, this is currently not the case for de-identified medical imaging^21–23^. This is already an area of active debate, as biometric identifiers of unique individuals continue to be developed, and many types of medical images are highly unlikely to be exactly homomorphic between individuals, particularly with today’s high resolution imaging and sophisticated image processing software. In the case of the eye, retinal vein patterns, iris patterns, and idiosyncratic eye movement patterns have all been shown to be accurate biometrics^24–26^. Meibography, given the highly detailed morphology of the Meibomian glands, could also serve as a biometric identifier with some further development of current technology. In this study, we have shown that a relatively straightforward deep learning algorithm trained on a fairly small dataset was capable of extracting some of the demographic characteristics of the subjects that provided the de-identified meibography images. Further development of the models and larger training datasets will certainly gain in accuracy and specificity, with individually identifiable meibography images on the horizon. While there is a potential for meibography’s use as a highly accurate biometric identifier, there is also concern with the lack of regulation and enforcement of the privacy and security of such images (assuming they are de-identified). Considering that combining data from multiple sources (e.g., multi-modal biometrics) significantly increases the likelihood of accurate individual identification^43,44^, it seems clear that urgent updates are needed in the regulations and enforcement governing the security of all biomedical imaging of patients and research subjects, regardless of whether it has been anonymized.

The study has certain limitations. Following a previous work^20^, only Meibomian gland morphology in the central upper eyelid was analyzed as imaging the central region of the tarsal plate with an optimal focus causes defocus of the peripheral glands. The decreased imaging quality of peripheral glands could lead to inaccurate analysis and thus only central regions were utilized. This work thus only presents results on demographic features and central tarsal plate morphological features as morphological features of peripheral glands were not used in this analysis. Additionally, images from lower eyelids were excluded in this study because gland features (e.g., % atrophy area) referencing to the lower tarsus area cannot be accurately quantified because the border of the lower tarsus is not accurately defined when eyelid eversion exhibits an exposed area that is larger than the lower tarsus^20^. It has also been reported that the border of the lower tarsus and the glands can be blocked by conjunctival folds^19^. For ethnicity, due to limited and imbalanced samples for some races, only Asian and Caucasian meibography images were distinguished using this deep learning approach. This leaves morphological features for other ethnic groups undiscovered.

In conclusion, an interpretable deep learning model to predict demographic characteristics from meibography images was developed. The model could be helpful in furthering the understanding of the relationships between local features of Meibomian gland morphology and subject demographics. Future work will extend the model to investigate detailed aspects of Meibomian gland morphology and the signs and symptoms of MGD and other types of DE. Finally, this work suggests that de-identified meibography images—currently not considered PHI and thus not subject to strict regulations on their use and dissemination—could in the future be developed into biometric identifiers of individuals with the rapidly evolving capabilities of artificial intelligence.

## Data Availability

The datasets used and/or analyzed during the current study are available from the corresponding author on reasonable request.
